# The potential of complementary and alternative medicine in promoting well-being and critical health literacy: a prospective, observational study of *shiatsu*

**DOI:** 10.1186/1472-6882-9-19

**Published:** 2009-06-18

**Authors:** Andrew F Long

**Affiliations:** 1 School of Healthcare, University of Leeds, Room 3.10, Baines Wing, Leeds, LS2 9UT, UK

## Abstract

**Background:**

The potential contribution of complementary and alternative medicine (CAM) modalities to promote and support critical health literacy has not received substantial attention within either the health promotion or the CAM literature. This paper explores the potential of one CAM modality, *shiatsu*, in promoting well-being and critical health literacy.

**Methods:**

Data are drawn from a longitudinal, 6 months observational, pragmatic study of the effects and experience of *shiatsu *within three European countries (Austria, Spain and the UK). Client postal questionnaires included: advice received, changes made 6 months later, clients 'hopes' from having *shiatsu *and features of the client-practitioner relationship.

**Result:**

At baseline, three-quarters of clients (n = 633) received advice, on exercise, diet, posture, points to work on at home or other ways of self-care. At 6 months follow-up, about four-fifths reported making changes to their lifestyle 'as a result of having *shiatsu *treatment', including taking more rest and relaxation or exercise, changing their diet, reducing time at work and other changes such as increased body/mind awareness and levels of confidence and resolve. Building on the findings, an explanatory model of possible ways that a CAM therapy could contribute to health promotion is presented to guide future research, both within and beyond CAM.

**Conclusion:**

Supporting individuals to take control of their self-care requires advice-giving within a supportive treatment context and practitioner relationship, with clients who are open to change and committed to maintaining their health. CAM modalities may have an important role to play in this endeavour.

## Background

There are a variety of ways to achieve health promotion's goal of increasing 'people's control over their health and its determinants' [1,2]. Within a population health approach, interventions would target not just strategies to enable healthier living and treatment of presenting symptoms, but also factors 'upstream', the primary causes of ill-health, within the wider socio-politico-economic environment in which people live [3,4]. In contrast, within routine health and medical care, interventions target individual patients, looking towards optimum strategies to promote and support individuals to modify their behaviour. Examples include use of motivational interviewing [5], applications of the stages of change model [6] and the notion of 'readiness to change' [7]. Other literature examines the manner in which treatment and care is provided, in particular, for example, shared decision making [8,9] and patient-centred care [10]. While (individual) patient empowerment [11] and self-efficacy [12] may be the ultimate goal, discussions focus on adherence to prescribed programmes and ways to support persons to manage their own ill-health. Left implicit, and thus outside the clinical gaze is the need to move beyond the individual, to consider the individual within their family and wider social network and socio-economic circumstances.

A central concept within health promotion is health literacy. Nutbeam's influential framework [13] differentiates three levels: functional health literacy (sufficient basic skills in reading and writing to function effectively); communicative/interactive health literacy (ability to extract information and to apply the information); and critical health literacy (ability to critically analyse information and to use it to exert greater control over life events). From a behavioural change perspective, critical health literacy is akin to a person heeding and acting on the advice given (after implicit or explicit reflection) and modifying their behaviour. Health literacy becomes an asset [14] and the intervention aims at enhancing individuals' control. The clinical or health promotion intention would be to move from providing information on, for example, healthy eating or healthier lifestyles (with an outcome focus on adherence with expert prescribed behaviour) to developing personal skills within a supportive (individual, family, community) environment (with an outcome focus on self-care in partnership with health care professionals) and onto individuals (and communities) taking control for self-care, with the self as the expert and manager [13,14]. Taking this a stage further, public health literacy embraces critical health literacy needed to make public health decisions that benefit the community [15] and effective actions at a political and social level to prevent ill-health or support health [13,16].

The potential contribution of complementary and alternative medicine (CAM) modalities to promote and support critical health literacy has not received substantial attention within either health promotion, CAM or the sociology of CAM literatures. Indeed, Hill [17] commented that UK health promotion texts rarely include reference to CAM and contain little in-depth discussion over their potential role in collaborative alliances to promote health. This is despite the increasing consumer user of CAM [18-20].

Core features of the philosophy and practice-based commitments of CAM and reasons for its use suggest a *prima facie *case for consideration of its role. Firstly, CAM modalities centre attention on health and healing. As Fulder [21] valuably enumerates, characteristics of alternative medicine include: restoring vital forces and self-healing energy (to awaken the immune system/response); working with, and not against, symptoms; seeking out the root of the problem [22]; exploring individualised paths for treatment; and, adopting a holistic approach to diagnosis and treatment. Secondly, characteristics of the practitioner-client encounter include: a more egalitarian relationship between client and practitioner in order to sustain and strengthen the client's commitment to taking (some) responsibility for health, well-being and self-care [23,24]; the practitioner listening [25] and providing a safe, 'protected' space [26]; and, seeing the presenting reasons/symptoms within the person's wider life and lifestyle [18,27]. The practitioner may act as teacher and consultant, as well as healer. Thirdly, while some access CAM to help to resolve a long standing condition [28,29], others talk explicitly about wanting to be proactive in order to prevent further ill health [30], engaging in active health maintenance and avoiding health-risk behaviours [29,31,32]. As Sointu [33] concludes from her analysis of interviews with practitioners and users of a variety of CAM practices, people may turn to CAM to 'seek a subjective sense of well-being rather than mere health .... The concept of well-being encapsulates a demand for being recognised as an active, empowered and knowledgeable agent' (pp. 345–346).

Such philosophical commitments find representation within the practice of CAM therapies. Energy medicine works 'more with what is felt than measured' [34]. Touching clients enables diagnosis, the delivery of the treatment and feedback as to how the treatment is received; it also creates a relationship between the practitioner and client. Classical (TCM) acupuncture's attempts to treat the 'root' (the underlying central disharmony) and 'branch' (the specific presenting symptoms) of the patient [22,35]. Some of the 'active ingredients' in homoeopathy, drawn out from case studies of individual packages of care by Thompson and Weiss [36] include the role of patient expectations (the expectation of potential benefit/belief in the treatment), openness to the mind-body connection, the expression of empathy within the consultation and the co-construction of the homoeopathic care, all apart from the remedy itself. More generally, in a study of CAM use, personality and coping strategies, Jacobson and Honda [37] suggest that 'openness to experience' may be a personality trait of persons who use body-mind, energy and other biologically-based CAM therapies. Finally, in the context of CAM education provision, Rakel *et al *[38] revisit the notion of salutogenesis, arguing for the necessity of health education to include a core understanding of healing and prevention. They illustrate their discussion through a review of evidence of CAM therapies for low-back pain. Areas of influence include mind-body, nutrition (sustaining food choices), spirituality (helping the patient to connect with things that give their life meaning) and the bio-energetic dimension.

Against this background, this paper seeks to explore the role of one CAM modality, *shiatsu*, to enhance critical health literacy and thus wider population health. *Shiatsu*, a body-based life-energy therapy, is a holistic health care method developed in Japan and influenced by Western knowledge. It is also inherently a safe modality [39]. *Shiatsu *uses Oriental energetic diagnosis and body energy techniques to correct imbalances in the body and focuses on the whole person, mind, body and spirit, as an interconnected whole, together with the environment in which the person lives [40]. All aspects of the client's life-energy system are addressed in understanding the condition, making an energetic diagnosis and giving a treatment. A highly developed sensitivity of touch enables the practitioner to feel and interpret the quality and flow of *ki*, the body's life-force. Treatment thus embraces both the application of gentle pressure to the energy channels on the body surface and commonly includes advice-giving, centred on raising self-awareness, modes of living and lifestyle to sustain good health. While there are many different styles of *shiatsu*, variations in theoretical content [40] and cultural dimensions surrounding its delivery [41], *shiatsu *training in Europe is grounded most commonly in the fundamentals of Traditional Chinese Medicine (TCM) philosophy and theory and by the approach of Shizuto Masunaga (Zen *shiatsu*).

## Methods

The study from which the data are drawn comprised a longitudinal, 6 months observational, pragmatic design of client experiences and effects of *shiatsu *as delivered and received in normal practice [42]. Following a pre-defined study protocol, clients were recruited by accredited and experienced *shiatsu *practitioners registered with one of three *shiatsu *national Societies: Österreichischen Dachverbands für Shiatsu (Austria); Asociación de Profesionales de Shiatsu de España (Spain); and the Shiatsu Society UK (UK). To be eligible for the study, practitioners had to be on the register for at least two years prior to the start date (Autumn 2005) and see an average minimum of 20 clients per month. Common characteristics of the training of the accredited practitioners included: part-time study over three years, with 500 to 700 teaching contact hours; similar energetic diagnosis and body energy techniques; supervised clinical practice; and, exploration of two of the three or four theoretical models of *shiatsu*.

All clients were 18 years or over and receiving *shiatsu *for whatever reason. Treatment was individualised for the client, often including advice-giving on lifestyle and other factors as well as direct energy-based bodywork. Data were collected by self-administered, postal questionnaires at four time-points: at initial ('baseline') recruitment, subsequent to the *shiatsu *session; four to six days after the recruiting *shiatsu *session; and, 3 and 6 months later. The content of the questionnaires was grounded in an interview-based, two country (Germany and the UK) study [32], exploring a range of *shiatsu-*specific and more general areas (Appendix 1). To provide data on factors associated with advice-giving, questions included: what clients 'hoped to get from having *shiatsu*' (at baseline), features of the client-practitioner relationship and advice-giving 'in the (recruiting) session', and changes made 'in their life as a result of having these *shiatsu *treatments' (at 3 and 6 months). If they had made any changes, they were asked to indicate in what area(s), choosing from a list of possibilities (for example, diet, exercise, rest and relaxation) and to describe 'any other changes' in the space provided.

A postal questionnaire was completed towards the end of the study by the practitioners who took part in the study to provide insight into how they practised *shiatsu*. This included a question on whether they commonly gave 'other advice and/or recommendations to the client' and to indicate in which areas, ticking from a list (for example, diet, points/meridians to work on at home, exercises, lifestyle habits and posture/how to use your body).

All data were coded and analysed using SPSS 13.0 for Windows. Data analysis was restricted to clients who completed all four study questionnaires in each of the three countries. As the study was hypothesis-forming, simple descriptive statistics are reported here. For the client written comments, a thematic approach was used, involving close reading and re-reading of the comments, identifying categories/themes to cover these, comparing categories and, finally, generating more abstract, theoretical labels [43]. Ethical approval for the study was obtained from the University of Leeds Faculty of Medicine and Health Research Ethics Committee.

## Results

### Participant Characteristics

Over an eleven month period (February-December 2006), 948 clients were recruited by 85 practitioners; 633 clients completed all four questionnaires, from baseline to six-month follow-up, an overall response rate of 67%. This varied from 49% (Spain, n = 93) to 70% (Austria, n = 261) and 72% (the UK, n = 279). A typical client was a woman (80–84%), aged in her 40s, in paid employment, either full- or part-time, who had used *shiatsu *before (84–88%) and described her overall health status as being 'good' or better. She was continuing to use *shiatsu *at three months (79–96%) and at six months (76–81%), having an average of 2–3 sessions during each three-month period. She also paid for her own treatment. There were some country variations. The UK sample was typically older on average (a median age of 50 years) and included a larger proportion of persons aged 65 and over (21% vs. 7–10% in Spain and Austria) and/or retired people.

Seventy-five practitioners completed the practitioner questionnaire, a response rate of 88%. A typical recruited practitioner was female and in her mid-40s, with formal education to at least Baccalaureate or A level standard, and was as likely to be working full- as part-time and involved in teaching *shiatsu *or not. She had been giving *shiatsu *for around nine years. Masunaga/Zen *shiatsu *on its own or in combination with TCM theory and practice was the most common practice style (84–89%). All the practitioners indicated that they commonly gave advice, where appropriate, relating to exercise, diet, lifestyle habits and/or posture or how to use one's body. There were a number of country differences. For example, Austrian practitioners on average had been in practice for 7 years compared to 12–13 years for the UK practitioners. UK practitioners were also more likely have a part-time *shiatsu *practice, to be involved in teaching *shiatsu *and least likely to be trained or qualified in another CAM therapy (although two-thirds were).

### Reasons for Accessing Shiatsu and Hopes from Treatment

At baseline, the main reason for accessing *shiatsu *'today', mentioned by 48% of respondents, was 'to maintain or improve their health.' When asked what they hoped to get from having *shiatsu *treatment, the second most mentioned was 'to enhance their health', quality of life or personal growth. The ways that these hopes were expressed were illustrative of a 'desire to change' (Appendix 2), and in many cases an implicit recognition of their own role in achieving change (for example, '*to get to know myself better*' S95; '*...a positive attitude*' UK237; '*... to know my body and its weak areas better so I can work on them*' S110; '*... to gain insights through connection*' A104; and, '*... with the exercises recommended*' UK200).

### Advice-Giving

At baseline, around three-quarters of clients indicated that their *shiatsu *practitioner gave them self-care advice or recommendations (Table 1). This picture was replicated by the practitioners; at least 80% indicated that they commonly gave such advice. 'Other' areas of advice included: ways to enable self-care, including stress management, self-massage, meditation, visualisation and use of herbal remedies (24%); emotional advice (21%) ('*about my attitude in facing life*' S56; '*make think about how I feel about myself*' UK98; '*positive attitude about my body ...mind*' A115); and preventive advice (16%) ('*pacing [myself] so as not to overtax my [body's] resources*' UK391; '*to pay attention to when my back is hurting*' S95; or, '*to listen to my own body, to look after myself more*' A258). Practitioners talked in similar terms, such as: '*(to) investigate pastimes that may fulfil (the) client's necessities*' (SP62) or '*meditation, growth work, emphasise their strengths and abilities*' (UKP14). The advice or recommendations were overwhelmingly perceived as relevant by the clients (99%).

**Table 1:** Areas of Advice Received and Given and Their Update

	**Austria**	**Spain**	**UK**
**Advice Received by Clients **^(1) ^(% yes)			

Advice or Recommendation given	76	76	74

Advice given in the following areas:			

- Exercise	65	48	48

- Diet	49	30	42

- Posture or how to use your body	27	51	29

- Points or meridians to work on at home	29	26	26

- Other	34	20	24

**Areas of Advice Given by Practitioners**^(2) ^(% yes)			

Exercise	94	100	96

Diet	87	83	96

Lifestyle habits	84	83	96

Posture or how to use your body	81	78	77

Points or meridians to work on at home	74	72	92

Recommend to consult another practitioner	65	89	85

Other	32	17	35

**Client Uptake of Advice**^(3)^			

Made lifestyle changes 'as a result of having *shiatsu *treatment' (% yes)	77	80	80

- Rest and relaxation (% take more)	75	80	54

- Exercise (% take more)	64	53	43

- Diet (% changed)	58	45	56

- Work (% reduce)	32	15	19

- Other (% yes)	33	48	40

**Key**: These data draw on three sources: (1) baseline client questionnaire; (2) practitioner questionnaire; and (3) six month follow-up client questionnaire

### Advice-Taking and Lifestyle and Awareness Changes

By six months follow-up, around four-fifths of the clients reported making changes to their lifestyle 'as a result of having *shiatsu *treatment' (Table 1). Substantial proportions had increased the amount of 'rest and relaxation' and 'exercise' they took (43–80%). Working less was also evident, interpretable from the verbatim comments in terms of 'time at work' or 'time devoted to work outside of work hours'. A third or more indicated making 'other' changes; the most mentioned areas were 'body/mind awareness' and changes in 'levels of confidence and resolve' (Appendix 3). Changes in self-perceptions over levels of confidence, levels of awareness and wider attitudes to health were reported; around two-thirds or more (64–87%) agreed or agreed strongly with statements about, for example, 'greater confidence', being 'more in touch with my emotions', changes in 'understanding and experience of my body' and 'more able to cope with things.' (Table 2)

**Table 2:** Self-Perceived Awareness Changes (% 'agree' or 'agree strongly' at six months follow-up)

	**Austria**	**Spain**	**UK**
**Overall Effects**			

I feel more confident about my health	87	80	79

**General Awareness**			

I feel more able to help myself	69	87	83
I am more in touch with my emotions	60	70	57
I think about things differently	68	64	63
My understanding and experience of my body have changed	82	66	72

**Attitudinal and Personal**			

I feel more hopeful that my problems can be helped	76	81	83
I am more able to cope with things	68	77	70
I feel I have developed as a person	67	61	52

### Features of the Client-Practitioner Interaction

Clients were overwhelmingly positive about their relationship with the practitioner. Around 70% or more of the clients 'strongly agreed' that their practitioner 'listened' or 'accepted' them (Table 3). While there was greater variation about their joint working, 75–93% expressed agreement that they did (30–46% 'strongly agreed'). The practitioner was also perceived by four-fifths of the clients as being 'trustworthy' and 'skilful' and as 'warm' by around two-thirds.

**Table 3:** The Client-Practitioner Relationship (% 'strongly agree'/% 'very much so')

**Client-Practitioner Relationship**	**Austria**	**Spain**	**UK**
The practitioner *accepted *me	76	68	72

The practitioner *listened *to me	84	70	69

The practitioner and I worked together*	46	30	34

			

I felt the *shiatsu *practitioner was *trustworthy*	89	87	86

I felt the *shiatsu *practitioner was *skilful*	86	82	81

I felt the *shiatsu *practitioner was *warm*	62	69	66

			

I liked the *treatment environment*	68	85	57

* These percentages increase to 92%, 75% and 85% respectively if % 'agree' is included

## Discussion

### Shiatsu and Advice-Taking

Examining these findings from a health literacy perspective suggests a valuable role for *shiatsu *in promoting healthier behaviours. At a basic, functional level, developing awareness and knowledge arose within advice-giving (diet, exercise, how to use your body and self-care) occurring in the baseline treatment session. It raised the possibility for the client to utilise this information in their everyday life. Such advice-giving occurred in the context of a client-practitioner consultation which was positively perceived by clients as involving 'listening' and 'accepting' the client and treatment by a skilful, warm and trusted practitioner. The fact that, six months later, around four-fifths of clients reported making substantial changes in their lifestyle 'as a result of having the *shiatsu *treatments' is indicative of their acting on the knowledge (interactive health literacy) and onto critical health literacy. Clients reported changes in exercise and diet, enhanced confidence about their health, being 'more able to help myself' and having a changed understanding and experience of their body. Overall, the lifestyle changes were suggestive of a tendency to adopt a more relaxed, healthier and more balanced approach to life.

One of the strengths of the study is its pragmatic nature, studying *shiatsu *as delivered and received in normal practice. While it is notable that the study findings were consistent across countries, there remains a possibility of inter- and intra-country variation, in particular in relation to the practitioners' style of practice. Despite similar training and use of Zen *shiatsu *on its own or in combination with TCM theory, practitioners will be at different stages in their own personal development, both within *shiatsu *and more broadly, leading to possible variations in how they practise. This is an important area for further research [44]. It is also important to note that the sample group were relatively socially and economically advantaged. The latter issue does not however affect the wider argument of the potential contribution of *shiatsu *to critical health literacy.

It is instructive to reflect on why *shiatsu *might result in so large a lifestyle change. At the level of theory, all presenting reasons, symptoms, responses during and after treatment would be understood and evaluated in terms of the person as an '*energetic*' being. Its holistic philosophy is enacted in the holistic nature of *shiatsu *practice, treating mind body and spirit as an interconnected whole together with the environment in which they find themselves. This concept of holism pervades the *shiatsu *encounter; energetic diagnosis includes questions pertaining to all aspects of a person's life. Clients' reasons for using *shiatsu *(health maintenance and health enhancement) and changes in lifestyle resonate with Antonovsky's concepts of a sense of coherence and generalised resistance resources [3,45]. Clients were seeking support in order to enable better health and living a good life, along with or as a consequence of, greater mind/body awareness.

A different explanation could relate to the nature of the client group *per se*. The typical user had chosen to access *shiatsu*, paid for by herself, had used *shiatsu *before and commonly was looking to maintain or improve her own health. From a social psychological, behavioural change perspective, the sample group was already motivated or ready to change and expectant of particular forms of benefit. At the same time, this may be only a part of the reason. Indeed, in-depth interviews with acupuncturists taking part in a pragmatic randomised controlled trial of acupuncture for low back pain drew attention to the integral role of self-help advice within the delivery of traditional acupuncture [46]. Other possible factors include the features of the client-practitioner interaction and the context and practice of care-giving. For example, studies within homoeopathy [47] and acupuncture [48,49] point to the importance of an empathic consultation and relational style of the practitioner, its influence on enabling the client, for example, to understand and cope with their illness, and a potential relationship with changes in perceptions of well-being.

At a methodological level, however, there is the possibility that the findings are an artefact, brought about by social desirability. Clients might have wanted to be supportive to the practitioner and thus were over-optimistic in their judgement of changes 'as a result of *shiatsu*'. It is however unclear why clients would not be honest, as typical users were continuing with *shiatsu*, it providing 'ongoing support' as one part of their approach to well-being and living healthily. Indeed, for the one or two clients who indicated that they had experienced 'a potentially adverse event or effect', none ceased using *shiatsu *[39]. Moreover, the study findings were consistent across the three countries [42]. Nevertheless, a social desirability effect remains a possibility. A future study might consider incorporating a specific measure to address this possibility [50].

### A Possible Explanatory Model for Research

**Figure 1:**
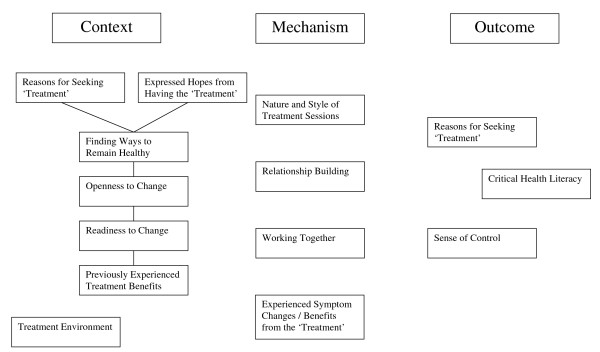
**C-M-O Configuration for Critical Health Literacy**.

To provide further insight into possible ways that a CAM could contribute to critical health literacy, self-care and promoting healthier behaviours, an explanatory model is presented in Figure 1. It is based on the logic of realist evaluation [51], in which causal outcomes (O) are seen as following from mechanisms (M) acting in particular contexts (C). The intention is to set up a number of plausible and testable hypotheses for further research both within and beyond CAM, drawing on the study's findings.

Three features of context are identified: client reasons for accessing the CAM, their expressed hopes from the treatment sessions and the treatment environment. Firstly, for this CAM, the main reason underlying why clients accessed it was 'maintaining or improving their health'. While the typical *shiatsu *user was in 'good' (or better) health, this is consistent with seeking help both to 'maintain health' and for pain or other symptoms. It also implies at least an interest in, if not commitment to, finding ways to remain healthy. Potentially implicit are notions of self-responsibility and an openness to change.

Secondly, the ways that the clients expressed their hopes for the treatments were highly suggestive of not just an openness to change [37], but also a readiness to change, for example, in terms of '*getting to know myself better*' or '*to know my body*.' As Dalton and Gottlieb [7] observe, readiness is linked to learning, and learning to empowerment, trying out new approaches and self-efficacy. In addition, many previous *shiatsu *users often linked their hopes to previously experienced benefits from earlier *shiatsu *treatments. It was also noticeable that the language used by some clients, including new *shiatsu *users, suggested a (growing) awareness of the theoretical underpinnings of an energy-based therapy, for example, talking in terms of '*to clear blockages*' or '*to become grounded*'.

Thirdly, the treatment environment plays an important contextual role, in terms of its ambience and atmosphere as well as features that may support its perception as a safe and caring environment [52,53]. The majority of the *shiatsu *clients strongly agreed that they 'liked' the treatment environment. Data from the practitioner questionnaires detailed how practitioners tried to make this environment a safe physical space (confidential and quiet) and a protected space (a 'no rush' treatment approach, a space for the client to talk, 'their space').

Possible mechanisms relate to the client-practitioner relationship and experienced benefit from the treatments; readiness to change is also important here retranslated as acting to change. The significance of the nature and quality of the client-practitioner relationship and relational style is widely reported in the CAM field as a therapeutic factor in achieved outcomes [47,48,54-56]. Initial perceptions of benefit, for example, in terms of symptom change and meeting prior expectations, is another possible mechanism, reflecting classic placebo theory [57,58]. Perceived benefits may become translated into ongoing self-care to maintain achieved (better) health and well-being. At the same time, experiencing positive symptom changes from treatment may not lead to changes in self-behaviour; the opposite effect could occur, as the 'problem' is seen to be resolved.

In summary, the model elucidates a set of factors that may account for, or be predictive of, seeking and taking advice. Engagement with the CAM modality, through an openness to change, taking responsibility for one's own health and enhancing potential control, coheres with the active realisation of critical health literacy by users. Thus, there are particular features of CAM (its philosophy and mode of practice), the way it is delivered (features of the client-practitioner encounter and environment of care) and characteristics of its users (seeking help, choice in access, openness and readiness to change) that interact to facilitate advice-taking and critical health literacy. While focus has lain on *shiatsu*, advice-giving is common within other CAM modalities, for example, acupuncture, ayurverdic medicine, herbal medicine, homoeopathy and traditional Chinese medicine.

The model suggests a number of areas for further research, in relation to *shiatsu *and other CAM modalities. Firstly, the C-M-O configuration requires further exploration, using measuring tools appropriate to each of its components. Secondly, it would be instructive to gain insight into how advice is actually delivered, who instigates it, effects on the relationship, especially the power dimension, and any possible variation by practice style. Thirdly, examination of the way that *shiatsu *and other CAM therapies might contribute to the client's sense of coherence and coping strategies, and its inter-linkage with critical health literacy, would be valuable in order to provide further evidence of the potential of CAM for population health. Fourthly, in this as in many other studies, reliance is placed on client reports. How perceived self-efficacy is in fact translated into lifestyle changes and/or whether or not individuals do in fact do what they say they do continue to be important areas to explore.

## Conclusion

This article has examined the role of CAM to enhance critical health literacy and health promotion, through a case study of *shiatsu*. The explanatory model arising has potential to aid understand of how critical health literacy may be enhanced both within and beyond CAM. The findings reinforce other research on the importance of an openness or readiness to change, advice-giving as part of an integral feature of some CAM practices and the continuing need for other initiatives around 'raising awareness' about healthy living, whilst exploring 'upstream' at an individual level, to their root and branch and wider socio-economic environment. At the least, the findings are strongly suggestive of a potentially powerful contribution of *shiatsu *to population health, particularly when used as part of ongoing support to maintain health, pursued on the initiative of users, albeit those who may be socially or economically advantaged.

## Key Points

• The potential contribution that complementary and alternative medicine (CAM) can make to promoting good health and developing critical health literacy is poorly understood

• Core philosophical and practice-based commitments of CAM modalities and reasons for its use suggest a prima facie case for consideration of their role

• Evidence from a large longitudinal, observational and practice study of one CAM, *shiatsu*, demonstrated both high rates of advice-giving and uptake six months later

• An explanatory model is developed to provide insight into why a CAM therapy could contribute to health promotion and enhanced health literacy and to guide future research both within and beyond CAM

## Competing interests

The author declares that they have no competing interests.

## Appendix 1

### Overview of Client Questionnaires

• *Questionnaire One – Baseline*: Socio-demographic characteristics of the client; previous use of *shiatsu*; how pay for *shiatsu*; reasons for use; severity of symptoms; use of other CAM and non-CAM for symptoms, use of medication and time-off work; hopes from *shiatsu *treatment; current health status

• *Questionnaire Two – Immediate Experiences and Effects*: experience, immediate positive effects and negative responses shortly after the recruiting *shiatsu *session; client-practitioner relationship; advice/recommendations given at initial session; immediate improvement; satisfaction with treatment; expectations met

• *Questionnaire Three – Positive and Negative Effects at Three Months*: continued use of *shiatsu*; symptom improvement, changed use of other CAM and non-CAM for symptoms, use of medication and time-off work; positive effects of having *shiatsu*; lifestyle changes; negative responses, if any; satisfaction with treatment; expectations met; current health status

• *Questionnaire Four – Positive and Negative Effects at Six Months*: continued use of *shiatsu*; symptom improvement, changed use of other CAM and non-CAM for symptoms, use of medication and time-off work; positive effects of having *shiatsu*; lifestyle changes; negative responses, if any; satisfaction with treatment; expectations met; current health status

## Appendix 2

### Hopes from *Shiatsu*: Illustrative Extracts for Enhancing Health

#### Physical and Psychological Health

Generally improved physical well-being, to become more balanced (tranquil). (A 281)

Improvement of health, physical and mental well-being. (A288)

To increase general well-being, control the physical complaints which are due to the mental problems according to my GP. (A100)

I hope to maintain my (physical and emotional) health, so I can get to know myself better. (S95)

To keep improving my physical condition, know myself better and improve my attitude in facing life and the world. (S94)

Having Parkinson's disease the *shiatsu *treatment helps slow down the progress of the disease, a positive attitude. (UK237)

#### Health in General

To improve my health, my tension, my back pain. To activate and harmonise my energy flow. (A171)

To maintain the level (of health/well-being) that I have reached through regular *shiatsu*. (A312)

To improve my health condition. To know my body and its weak areas better so I can work on them in order to improve. (S110)

To maintain general well being. To nip in the bud any potential stresses that could escalate if left untreated. (UK195)

I go once a month to keep me healthy and in a positive frame of mind. (UK238)

#### Personal Development

Enrichment in relation to self development. To gain insights through connection: emotion and body. (A104)

I want to do something for myself, to increase my capability for relaxation, to improve my health and sense of well-being. (A177)

#### Symptom Specific

To have less tension, especially in the back. Better well-being. (A234)

I hope to continue having good physical health, controlling problems with muscles, joints. (S3)

Keep physical problems at bay and keep symptoms to minimum ...some personal space and time for me to relax (UK209)

## Appendix 3

### Illustrative 'Changes' Extracts: Mind-Body Awareness and Confidence and Resolve

#### Mind/Body Awareness

Paying more attention to my body and its signals ... trusting in my senses. (A23)

When it comes to physical complaints I am more aware and take more responsibility. (A8)

More body awareness and more mindfulness, therefore more resting phases. (A22)

It reminds me that I am a living body and I am in control of my body. (S128)

I am more self-aware, I am more in touch with my emotions. I am more positive. (S138)

More body awareness which allows me to take better care of myself. (S186)

An overall awareness of what impacts my mind and body, negative and positive. (UK15)

I have developed a keener awareness of how thoughts and feelings are connected to and express themselves through the body. This awareness allows me to be more self sustaining through my healing process. (UK181)

#### Levels of Confidence and Resolve

Change in my self-image. (A154)

I'm better able to draw boundaries in my life. (A80)

Self-awareness, more courageous, more determined. (A210)

I regained control over my life; it's not my "knees" anymore. (A142)

Better control of emotions, greater self-reliance, fewer external needs. (S6)

Increase of self-confidence and awareness of my emotions. (S30)

My frame of mind has improved. (S45)

My ability to assimilate changes in my life, like situations where I do not feel comfortable, is more positive. (S129)

I'm changing my way of living. *Shiatsu *has opened that door to me. (S86)

Feel a greater sense of direction and what I want to do with the rest of my life. (UK110)

Ability to relax and approach problems more positively. (UK301)

Have learnt to like/respect myself for the first time. Pace myself. Aware of needs. (UK70)

Feel a lot more confident, lost a lot of weight, became a stronger person. (UK98)

I am much more content. I do not get so "up tight" about irrelevant things. I now am qualified to do Indian head massage plus *reiki*. My perspective on life has altered. (UK224)

## Pre-publication history

The pre-publication history for this paper can be accessed here:


